# A case report of using nivolumab for a malignant melanoma patient with rheumatoid arthritis

**DOI:** 10.1007/s13691-016-0256-8

**Published:** 2016-07-23

**Authors:** Shun-Ichiro Kageyama, Shigeo Yamaguchi, Shin Ito, Yoshiyuki Suehara, Tsuyoshi Saito, Keisuke Akaike, Kayo Miura, Shunsuke Kato

**Affiliations:** 1 Department of Clinical Oncology, Juntendo University Graduate School of Medicine, 2-1-1 Hongo, Bunkyo-ku, Tokyo, 113-8421 Japan; 2 Department of Otorhinolaryngology, Juntendo University School of Medicine, 2-1-1 Hongo, Bunkyo-ku, Tokyo, 113-8421 Japan; 3 Department of Orthopedic Surgery, Juntendo University School of Medicine, 2-1-1 Hongo, Bunkyo-ku, Tokyo, 113-8421 Japan; 4 Department of Human Pathology, Juntendo University School of Medicine, 2-1-1 Hongo, Bunkyo-ku, Tokyo, 113-8421 Japan

**Keywords:** PD-1, Autoimmune disease, Melanoma, Nivolumab

## Abstract

The use of antibodies against programmed cell death 1 (PD-1), which block inhibitory T cell checkpoints, is a promising new therapy for advanced malignant melanoma and NSCLC. However, patients with autoimmune diseases were excluded at the clinical trial using such immune checkpoint inhibitor, because of the possibilities to worsen an adverse event of the autoimmune disease. Thus, the efficacy and toxicity of nivolumab using such cases have not been reported yet. A 70-year-old woman with bone and duodenal metastasis of primary mucosal melanoma with complications of the rheumatoid arthritis was treated with nivolumab. After 4 weeks injection of nivolumab, bone metastasis was diminished. After receiving six courses of nivolumab therapy, she maintained a complete response for 9 months, without rheumatic exacerbation or drug-related adverse events. Establishment of the biomarker of the effect prediction of the PD-1 antibody, the adverse event prediction will be important in future.

## Introduction

Programmed cell death protein 1, also known as PD-1, is a cell surface receptor and is expressed on T cells and pro-B cells. PD-1 negatively regulates T cell activation when it binds to PD-L1 and PD-L2, which are over expressed on cancer cells. Blockading the PD-1/PD-L1 pathway could reverse the tumor microenvironment and enhance the endogenous antitumor immune responses. Nivolumab is a humanized IgG4 anti-PD-1 monoclonal antibody. Nivolumab has shown benefits in clinical trials of advanced malignant melanoma and NSCLC. However, patients with autoimmune diseases were excluded at the clinical trial because of the risk of autoimmune-related adverse events and the efficacy and toxicity of nivolumab using such cases have not been reported yet. Here, we report a case of a notable response to nivolumab administration in a patient with malignant melanoma and active rheumatoid arthritis during treatment.

## Case report

**Fig. 1:**
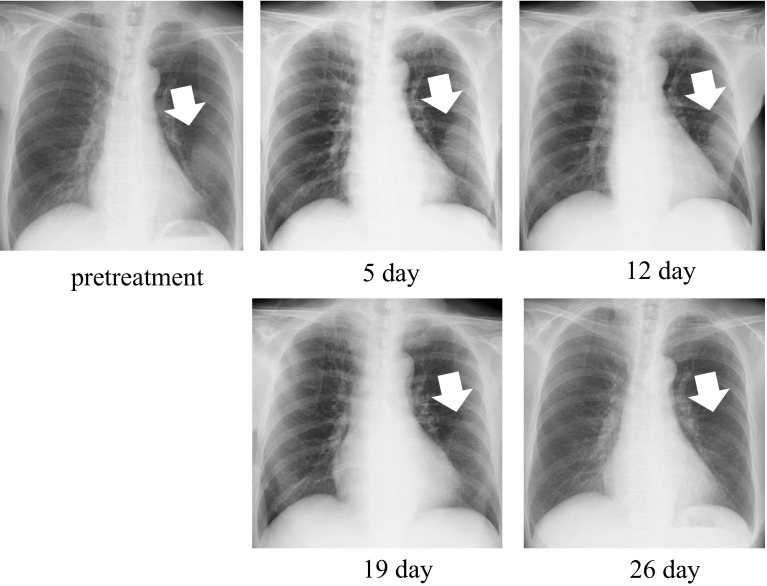
Response of a primary mucosal melanoma metastasis to nivolumab after the first treatment. The pretreatment chest radiographic image shows rib metastasis. Meanwhile, the chest radiographic image obtained 5–26 days after the first treatment shows that the costal tumor (*arrow*) had resolved

**Fig. 2:**
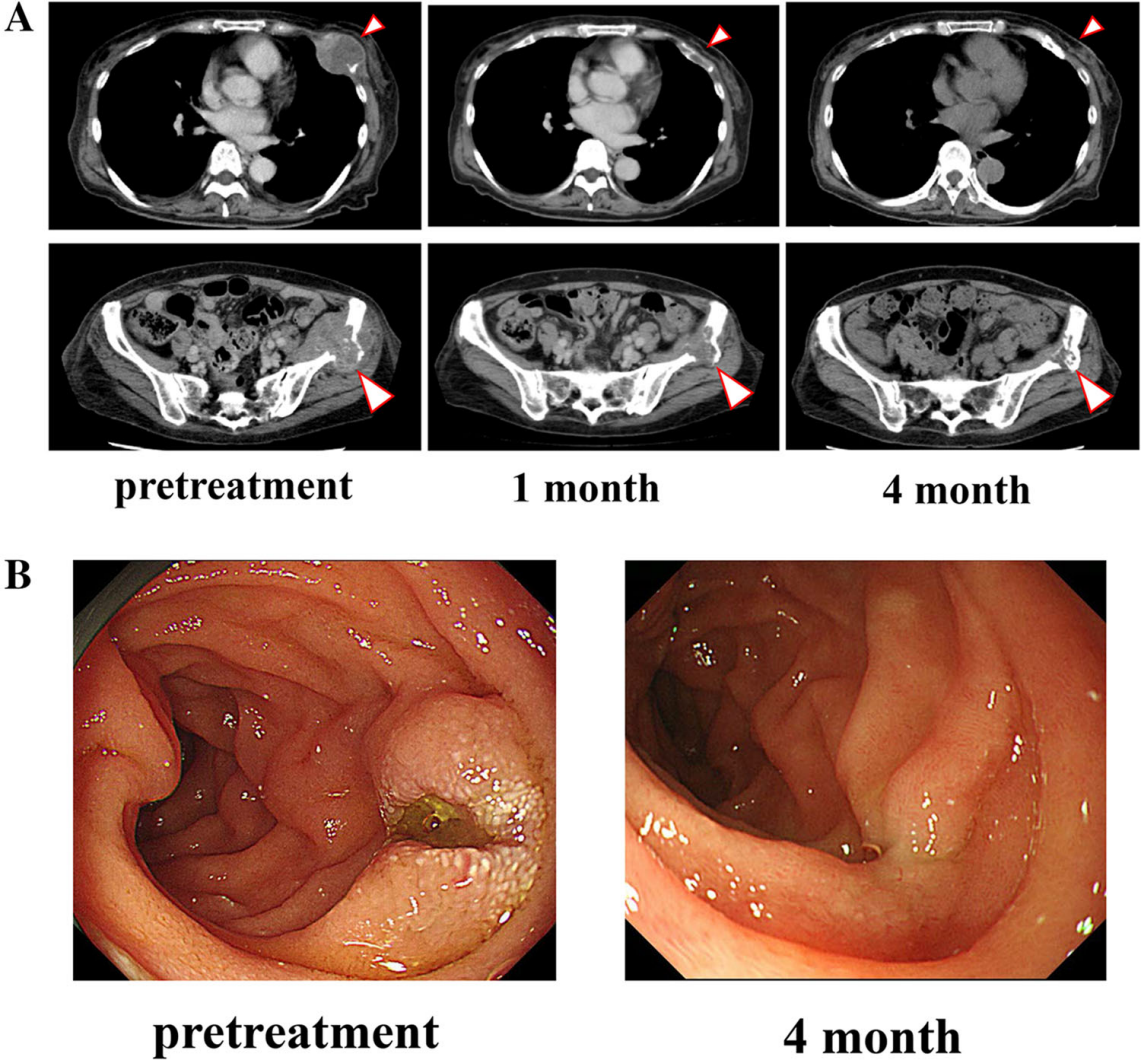
Demonstration of treatment response. **a** Computed tomographic (CT) scan with soft tissue windows of the bone metastasis. The rib and ileal lesions show acceptable reductions 1 month after the first treatment and nearly CR state was continued after 4 month. **b** The endoscopic image before and 4 month after the treatment

**Fig. 3:**
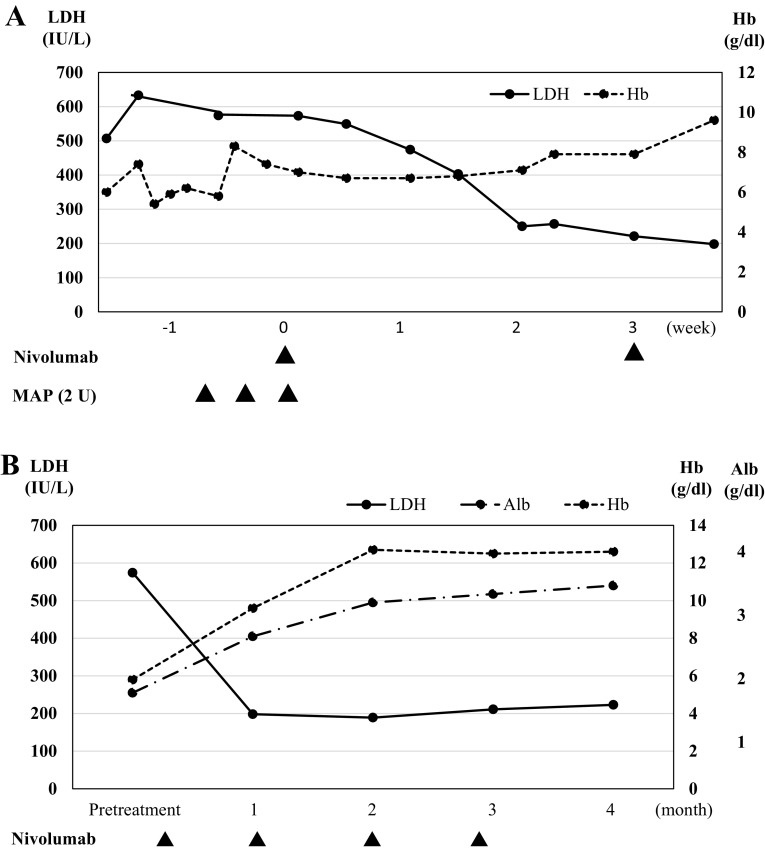
Nivolumab treatment course of patient with RA. Progress of laboratory data in first one month (**a**) and after 4 months (**b**)

A 70-year-old woman with active rheumatoid arthritis, who was under treatment with salazosulfapyridine 1,000 mg/day, was diagnosed as having BRAF wild-type primary mucosal melanoma. The melanoma was resected, and 60-Gy/30-Fr radiotherapy was administered as the adjuvant treatment. One year after the initial presentation, relapse occurred in the left sixth rib and left iliac fossa. Duodenal metastasis was also observed, wherein ulceration with gastrointestinal bleeding was identified. The patient had anemia and required transfusion at least three times per week. The patient received the first treatment session with nivolumab (2 mg/kg); no substantial adverse effect was observed. After 19 days, her rib tumor started to decrease in size. After 26 days, the tumor could not be visualized on chest radiography (Fig. 1) and no substantial adverse effects were observed. Computed tomography (CT) performed 1 month after therapy initiation showed the absence of the costal lesion and an acceptable reduction of more than 60 % of the ileal lesion (Fig. 2a). The ulcerated duodenal lesion on the endoscopy performed 4 months after the treatment showed cicatrization (Fig. 2b). Anemia due to bleeding from the tumor was reduced after the second week, and blood transfusion was discontinued (Fig. 3a, b).

**Fig. 4:**
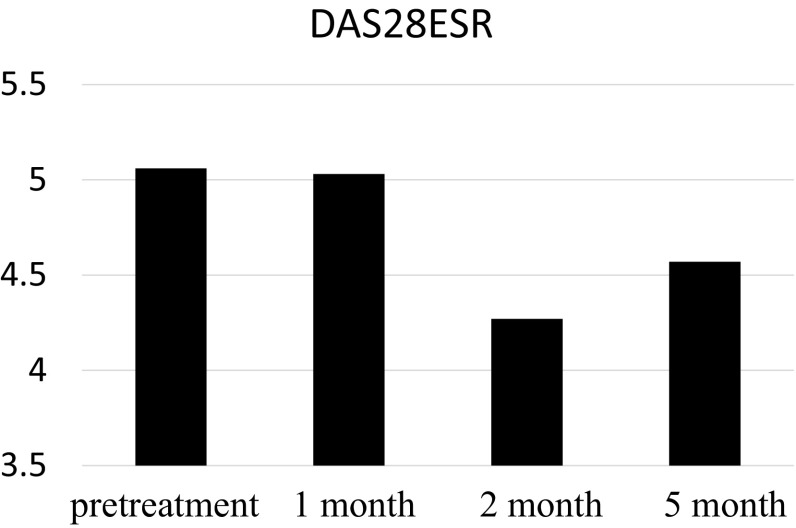
Evaluation of the activity of the rheumatoid arthritis from for treatment after treatment (*ESR* erythrocyte sedimentation rate)

During the four courses of nivolumab treatment, slight changes of the laboratory data were observed, however, there were no adverse events, and the joint pain and DAS28ESR scores did not get worse (Fig 4; Table 1). After receiving six courses of nivolumab therapy, she maintained a complete response for 9 months, without rheumatic exacerbation or drug-related adverse events.

**Table 1:** The activity of the rheumatoid arthritis

	Pretreatment	After 1 month	After 3 months
ANA	1280	1280	
DNA/RIA (IU/ml)	2	2	2
MMP-3 (IU/ml)	341.8	820.2	432.6
CH50 (U/ml)	36.5	35.3	41.5
C4 (U/ml)	14	18	25
C3 (U/ml)	74	94	98
IgG (mg/dl)	909	1457	1578
ESR (mm/h)	59	109	48

## Discussion

We describe a patient with active rheumatoid arthritis who underwent treatment with nivolumab immunotherapy for progressive melanoma. In a past clinical trial, the complete response rate for nivolumab was 3.3–8.9 % [1, 2]. Even in cases with the earliest onset of tumor reduction, the tumor took longer than 10 weeks to resolve. A recent case report described prompt complete responses to nivolumab and ipilimumab after interferon treatment [3]. However, the present case showed a rapid response, and at least one target lesion was diminished after a single course of treatment.

Immunne checkpoint inhibitors cause adverse events, including exacerbation of existing autoimmune disease such as myasthenia gravis, interstitial pneumonia, and thyroiditis. Hence, patients with autoimmune diseases were excluded at the clinical stage of the safety test [4].

Therefore, cases treated with PD-1 antibody therapy have not been reported, except a few cases of CTLA-4 antibody therapy for autoimmune disease (Table 2) [5]. The efficacy and adverse events in autoimmune disease patients treated with CTLA-1 antibody are seem to be higher than the results of the phase III clinical trials conducted excluded autoimmune disease so far (Table 2) [6].

**Table 2:** Past reports of the immune checkpoint inhibitor treatment for the autoimmune disease patients [5] and without autoimmune disease cohort [6]

	irAE		Response		
	Grade 3, 4	Grade 5	CR	PR	SD
Douglas et al. [5]					
Autoimmune disease	30 % (9/30)	3.3 % (1/30)	3.3 % (1/30)	16.7 % (5/30)	10 % (3/30)
(Rheumatoid arthritis)	40 % (2/5)	0 % (0/5)	20 % (1/5)	40 % (2/5)	0 % (0/3)
Hodi et al. [6]					
Ipilimumab + gp60	10.3 %	0.0 %	0.2 %	5.5 %	14.4 %
Ipilimumab	14.5 %	0.0 %	1.5 %	9.5 %	17.5 %
Total	11.3 %	1.3 %	0.6 %	6.5 %	15.1 %

The immediate effect observed in our case was also better than that of a single immune checkpoint inhibitor previously reported. Similar cases should be accumulated to further investigate the validity of the dosage for patients with autoimmune diseases. The primary finding in this study is the strong antitumor effect observed in the absence of exacerbation of existing autoimmune disease.

Rheumatoid arthritis is a condition where the cell-mediated immunity against self-derived antigens is activated. It is reportedly caused by several immune-related gene abnormalities [7], and the target gene of the immune checkpoint inhibitor is included in these genes. It is interesting that this meta-analysis and other studies report that CTLA-4 dysfunction (SNP splice abnormality) is related to rheumatism and that the functional recovery of CTLA-4 can be a treatment [8, 9]. As for the effect of the PD-1 antibody in this case, it appeared that SNPs of autoimmune-related genes such as CTLA-4 reinforced the antitumor effect of nivolumab, as combined therapy with nivolumab and ipilimumab yielded synergistic effects [1, 2]. The immune checkpoint inhibitor raises useful results, but establishment of the biomarker and autoimmunity-related adverse event management are future problems.

For the development of an immune checkpoint inhibitor, elucidation of autoimmune-related genes and such pathways is highly important and warrants future studies.
